# Supplementary material for: Design, Synthesis, and Evaluation of a Set of Carboxylic Acid and Phosphate Prodrugs Derived from HBV Capsid Protein Allosteric Modulator NVR 3-778
Source: Molecules. 2022 Sep 14;27(18):5987. doi: 10.3390/molecules27185987 (PMC9505734; doi:10.3390/molecules27185987)
Supplement: Supplementary file 1 [file molecules-27-05987-s001.zip › molecules-1903088-supplementary.pdf]

Supporting Information for

Original article

Design, Synthesis, and Evaluation of a Set of Carboxylic Acid and Phosphate Prodrugs Derived from HBV Capsid Protein Allosteric Modulator NVR 3-778

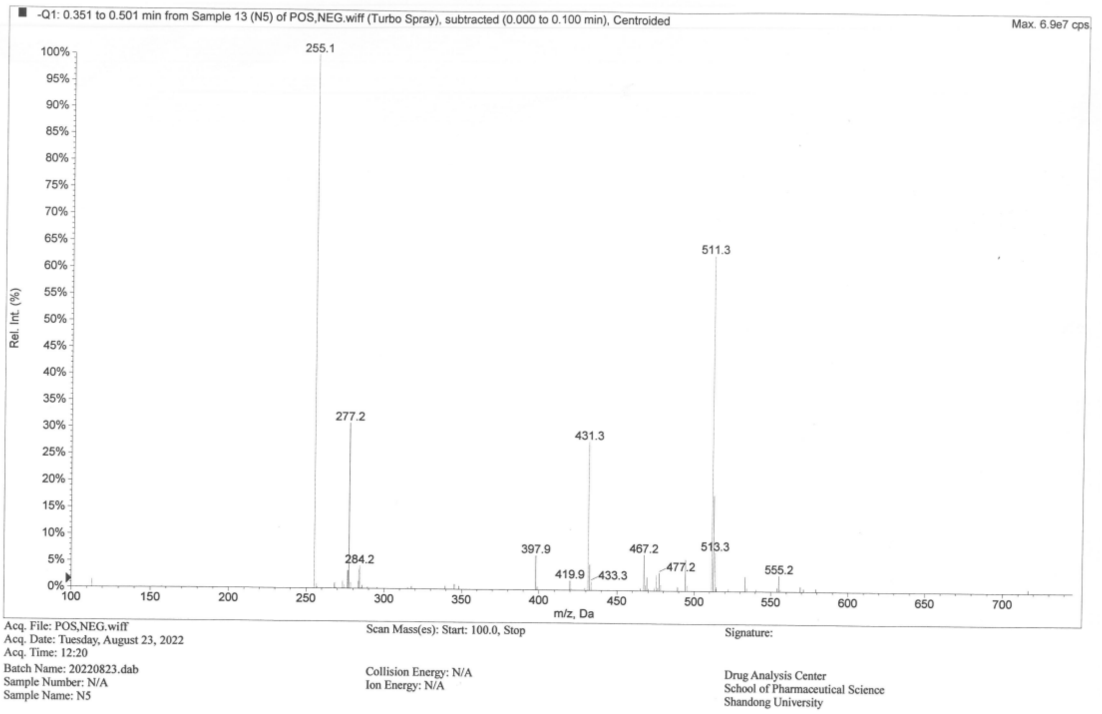

Mass spectrum of N5

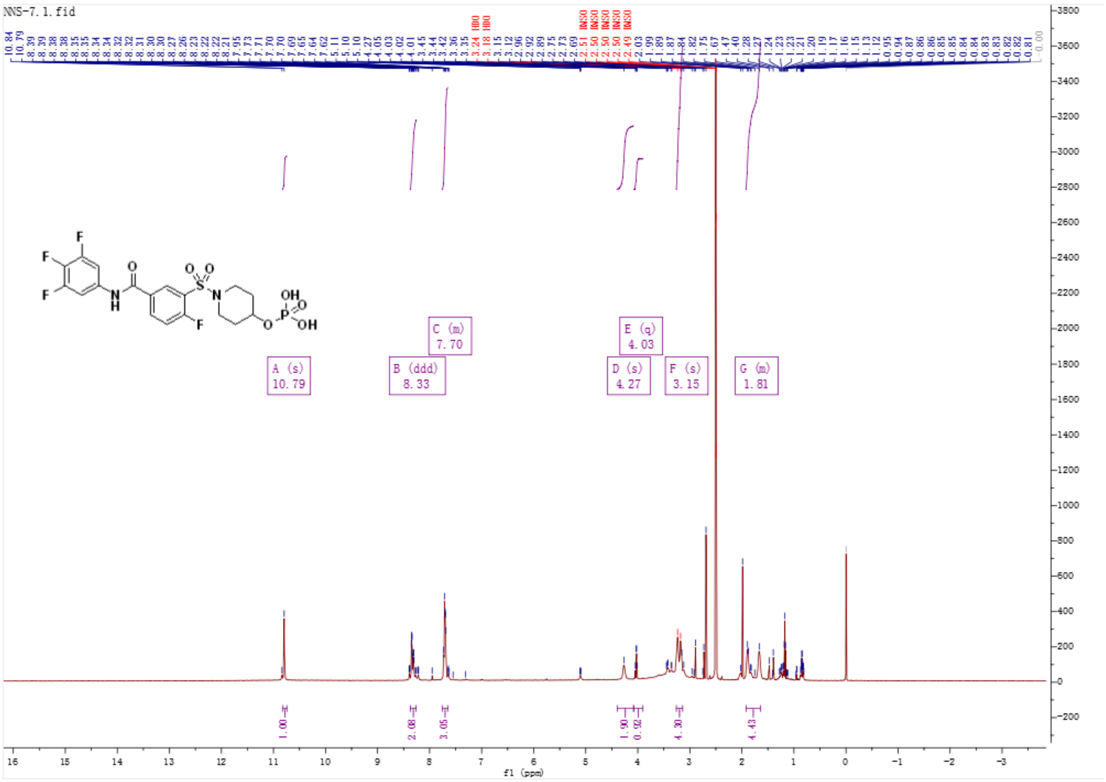

^{1}H NMR spectrum of N5

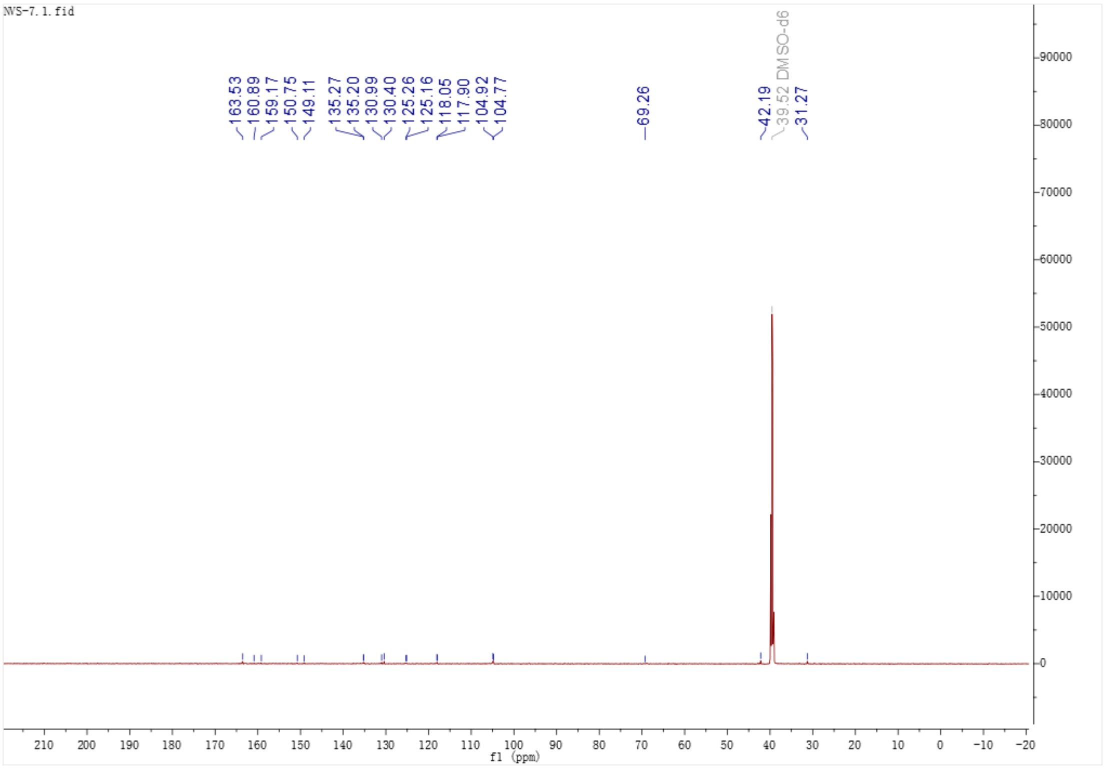

^{13}C NMR spectrum of N5

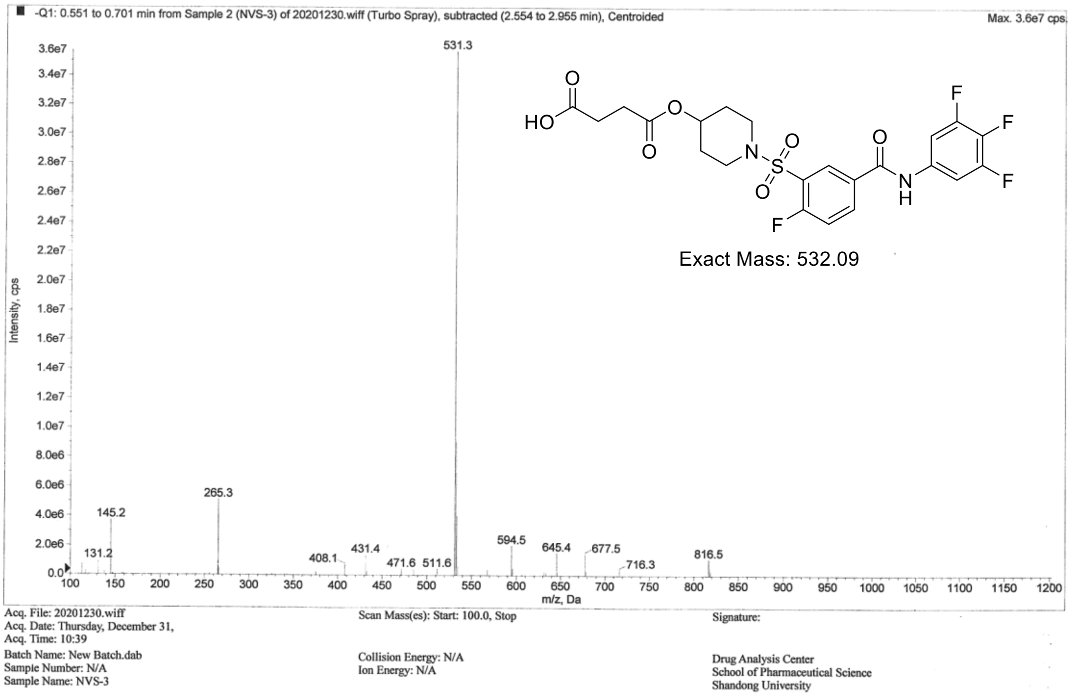

Mass spectrum of N6

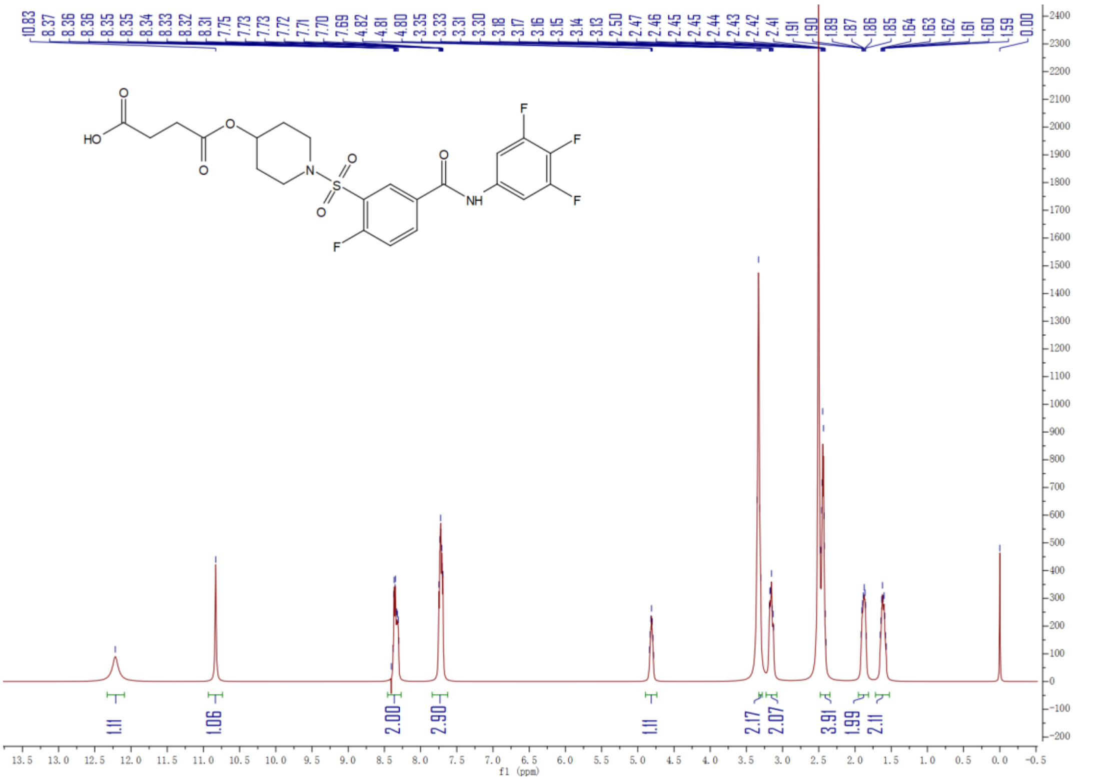

^{1}H NMR spectrum of N6

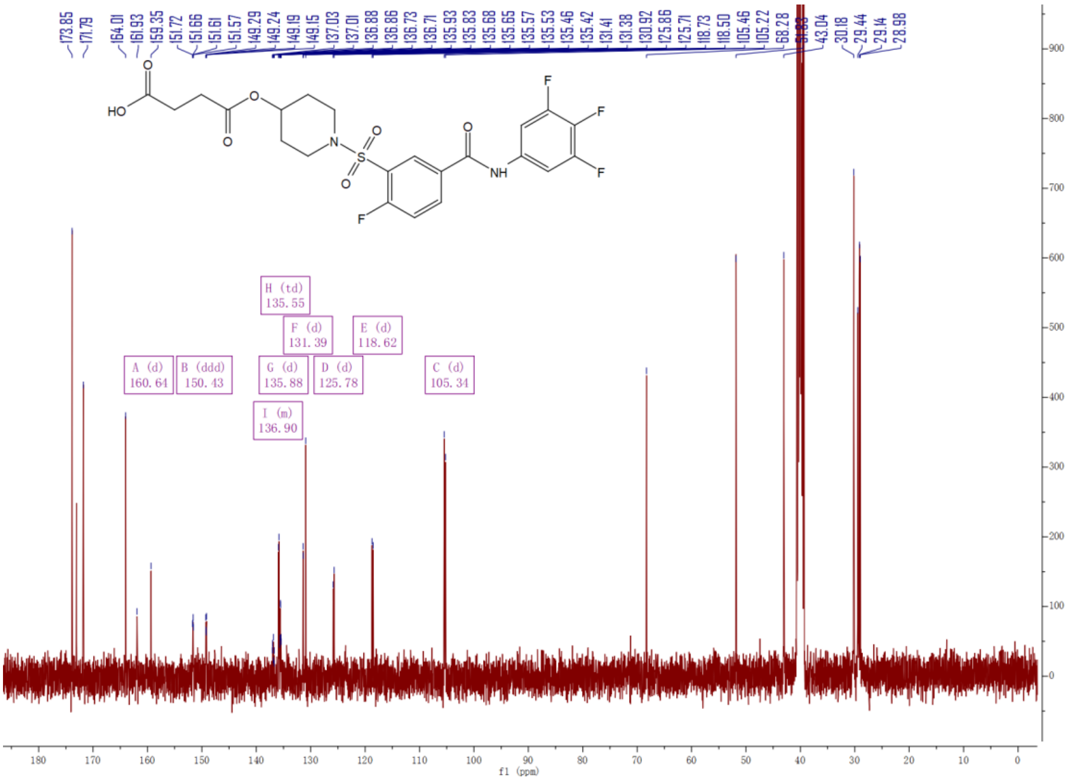

^{13}C NMR spectrum of N6

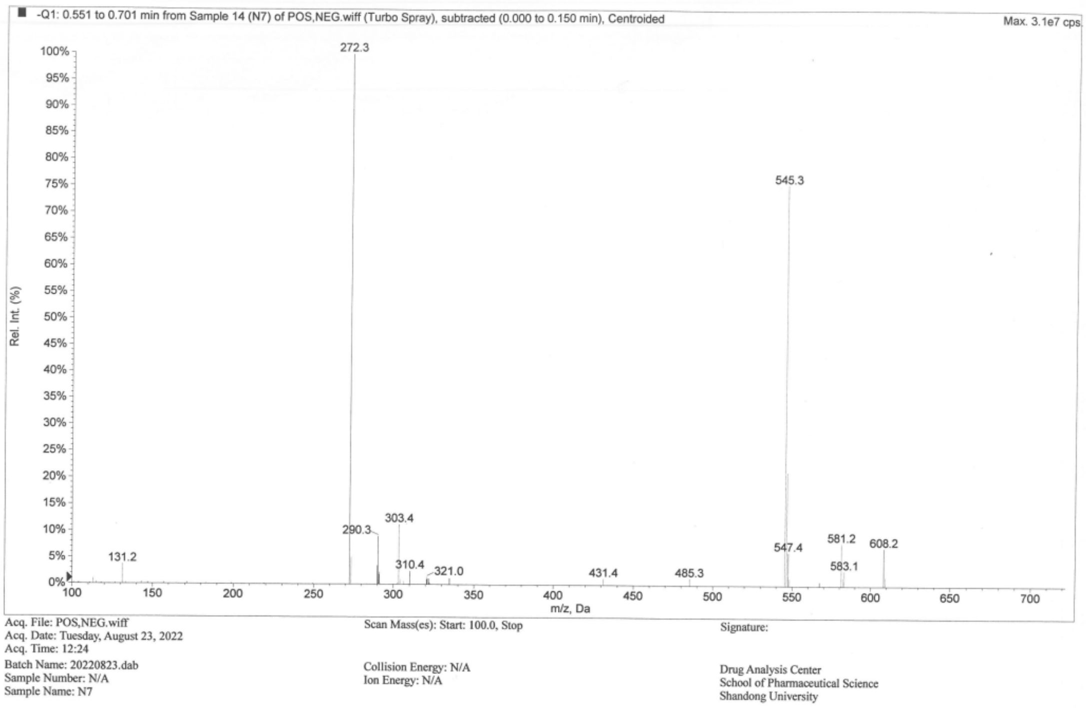

Mass spectrum of N7

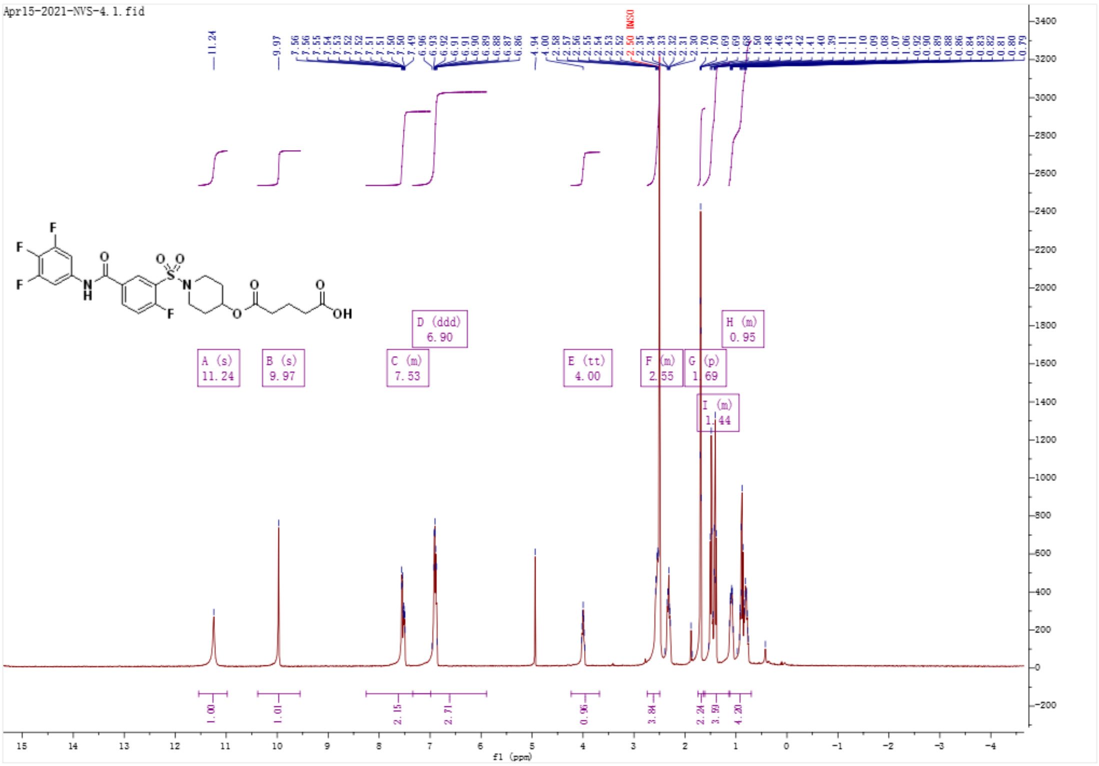

^{1}H NMR spectrum of N7

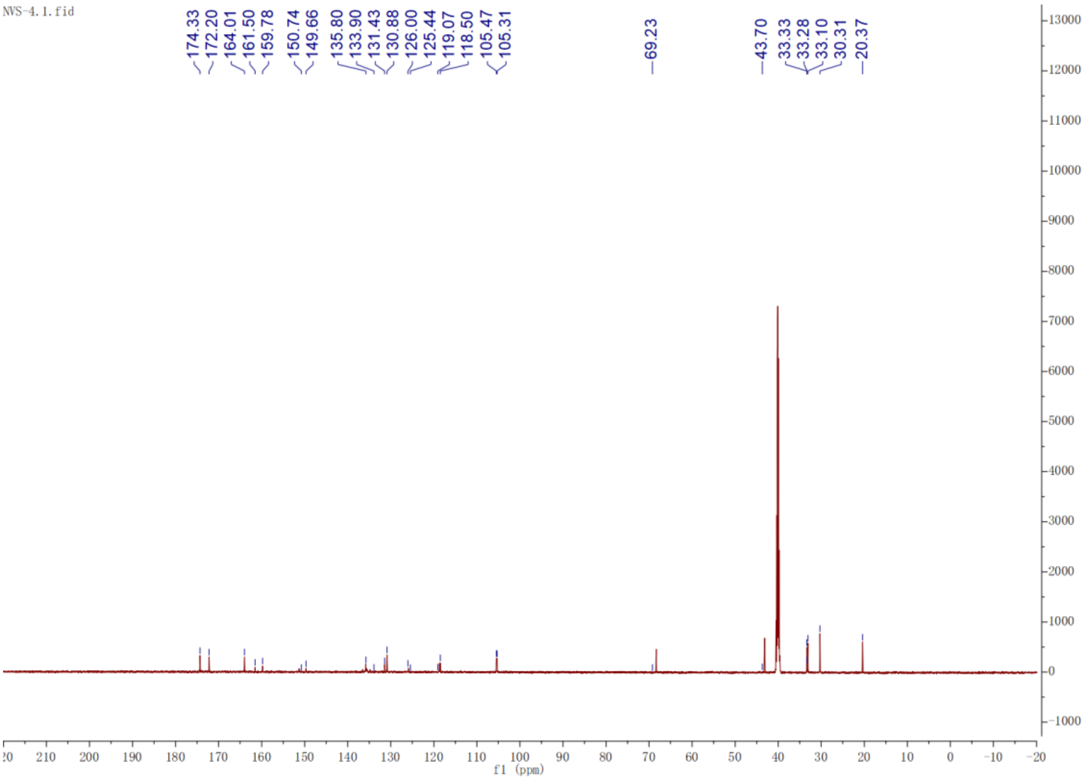

^{13}C NMR spectrum of N7

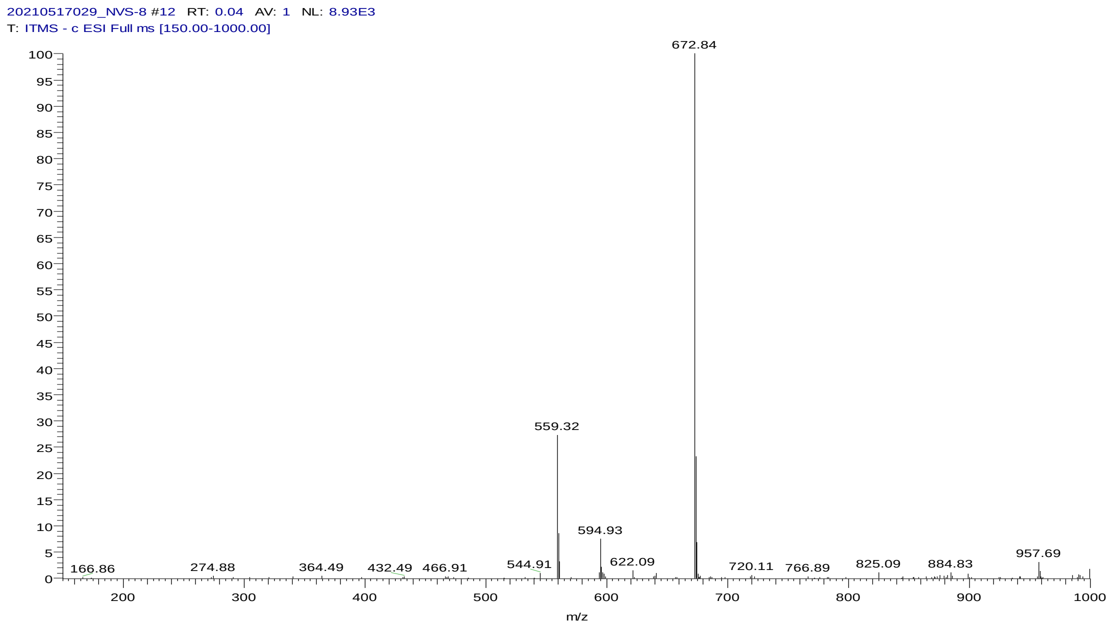

Mass spectrum of N8

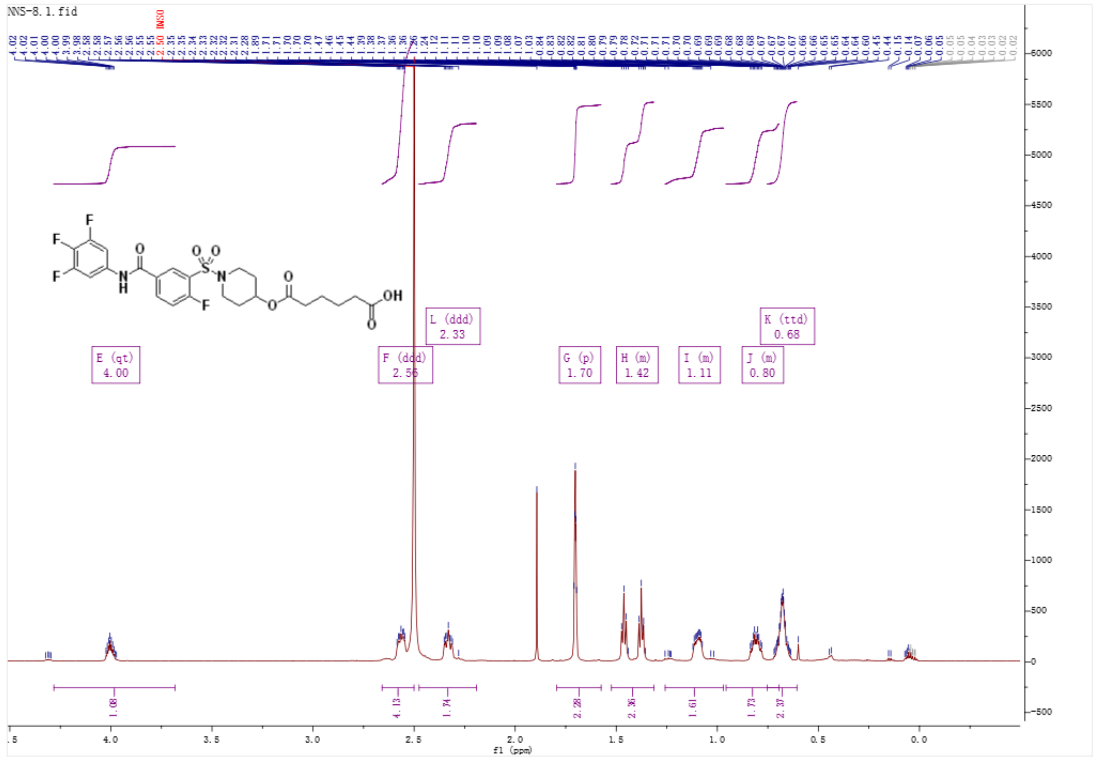

^{1}H NMR spectrum of N8

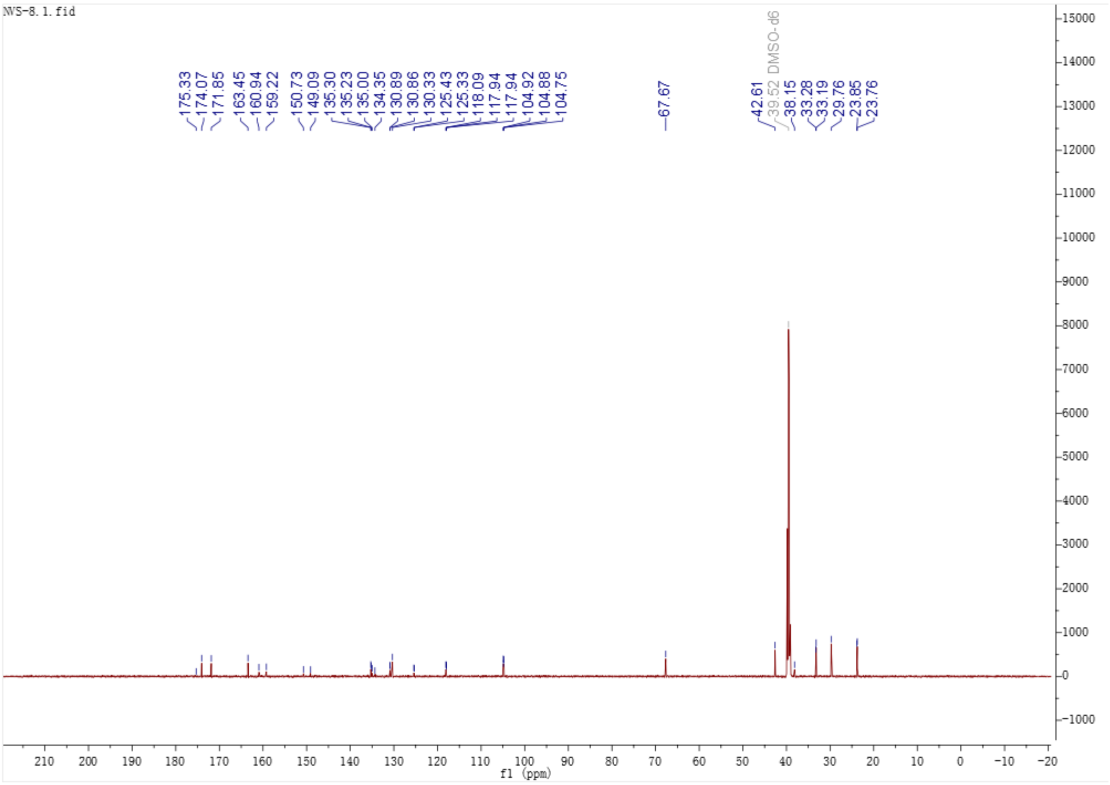

^{13}C NMR spectrum of N8
